# The First Paragraph of *the Organon*

**Published:** 1892-07

**Authors:** 


### THE FIRST PARAGRAPH OF *THE ORGANON.*

EDITOR OF THE HOMŒOPATHIC PHYSICIAN :

Will you kindly allow me space for a quotation, which will emphasize and enforce the teaching of your editorial in the March number ?

I have a friend—an M. D. of the old school—he is a graduate of one of the best, if not *the* best allopathic college in the United States. For the past two years he has been studying abroad. Three or four months ago, while at Vienna, he wrote : " The manner in which they [the physicians] diagnose the most obscure diseases here is something simply wonderful to me; but therapeutics seem to count but little; in fact, they seem disappointed if they are unable to verify their diagnosis by an autopsy."

Comment is unnecessary; this quotation is quite enough to show the necessity of keeping constantly in mind the first paragraph of *The Organon*, which is the sole *raison d'être* of the medical profession. A LAYMAN.

